# Validation that miRNAs altered in Alzheimer’s disease in human cerebrospinal fluid also function as miRNA biomarkers for Alzheimer’s disease in human plasma

**DOI:** 10.1002/alz.094980

**Published:** 2025-01-09

**Authors:** Julie A Saugstad, Ursula S Sandau, Jack Wiedrick, Trevor J. McFarland, Douglas R. Galasko, Joseph F Quinn

**Affiliations:** ^1^ Oregon Health & Science University, Portland, OR USA; ^2^ University of California, San Diego, La Jolla, CA USA

## Abstract

**Background:**

Alzheimer’s disease (AD) is the most common cause of dementia, and the fifth leading cause of death for those 65 and older. Brain changes in AD begin 10‐20 years before symptoms appear, yet markers for early brain changes are lacking. We discovered and validated miRNAs in human cerebrospinal fluid (CSF) that differentiate AD from Controls. However, as blood is a preferred biofluid for biomarker profiling, we evaluated 1) the intraindividual longitudinal stability of human miRNAs in plasma, then 2) expression of the CSF AD miRNAs in plasma.

**Method:**

For the longitudinal stability study, blood was collected by venipuncture biweekly over 3 months from 22 donors who had fasted overnight. For the CSF‐plasma miRNA correlation, we obtained 320 donor‐ and date‐ matched CSF and plasma AD and Control samples from the AD Neuroimaging Initiative. We isolated total RNA from 1) 200 µL of platelet‐free plasma or 2) 250 µL of CSF and plasma. MiRNA expression was assessed by qPCR using custom TaqMan human arrays. All studies were approved by OHSU IRB00009707.

**Result:**

1) For the longitudinal stability study, of 134 miRNAs amplified, 74 had high test‐retest reliability and low percentage level drift. Importantly, 13 candidate miRNA biomarkers for AD were stable in plasma over 3 months, and we found that hemolysis and tobacco use had the greatest impact on miRNA levels and variance. 2) For the CSF‐plasma study, the data revealed that individual plasma miRNAs are more predictive than CSF for AD, and a combination of plasma miRNAs are more predictive for AD than CSF. In addition, the highest‐probability plasma model for AD contains 2 complementary miRNAs, one positive (increased log fold change in expression), and one negative for fold change (decreased log fold change in expression).

**Conclusion:**

These studies revealed plasma miRNAs that are stable over the 3‐month time, including candidate AD biomarkers. They also revealed miRNAs that are not stable over the 3‐month time, including miRNAs previously reported as biomarkers AD. In addition, the CSF‐plasma studies validated that AD CSF miRNAs differentiate AD from Controls in plasma, supporting the use of plasma for further development of AD miRNA biomarkers for clinical assays.

